# The subcortical brain regions influence the cortical areas during resting-state: an fMRI study

**DOI:** 10.3389/fnhum.2024.1363125

**Published:** 2024-06-26

**Authors:** Omid Moazeni, Georg Northoff, Seyed Amir Hossein Batouli

**Affiliations:** ^1^Department of Neuroscience and Addiction Studies, School of Advanced Technologies in Medicine, Tehran University of Medical Sciences, Tehran, Iran; ^2^Mind, Brain Imaging and Neuroethics Research Unit, The Royal’s Institute of Mental Health Research, University of Ottawa, Ottawa, ON, Canada; ^3^BrainEE Research Group, Tehran University of Medical Sciences, Tehran, Iran

**Keywords:** fMRI, causality, effective connectivity, networks, resting-state, mind

## Abstract

**Introduction:**

Numerous modes or patterns of neural activity can be seen in the brain of individuals during the resting state. However, those functions do not persist long, and they are continuously altering in the brain. We have hypothesized that the brain activations during the resting state should themselves be responsible for this alteration of the activities.

**Methods:**

Using the resting-state fMRI data of 63 healthy young individuals, we estimated the causality effects of each resting-state activation map on all other networks. The resting-state networks were identified, their causality effects on the other components were extracted, the networks with the top 20% of the causality were chosen, and the networks which were under the influence of those causal networks were also identified.

**Results:**

Our results showed that the influence of each activation component over other components is different. The brain areas which showed the highest causality coefficients were subcortical regions, such as the brain stem, thalamus, and amygdala. On the other hand, nearly all the areas which were mostly under the causal effects were cortical regions.

**Discussion:**

In summary, our results suggest that subcortical brain areas exert a higher influence on cortical regions during the resting state, which could help in a better understanding the dynamic nature of brain functions.

## 1 Introduction

The human brain mapping studies have mostly relied on one of the two functional brain organization principles: functional segregation or functional integration. Functional segregation is based on the idea that spatially specific neuronal structures, such as certain brain regions, are responsible for processing the target functions, whereas functional integration refers to dispersed interactions among regions that are functionally distinct. Functional integration research seeks to understand how connections across brain regions govern regional responses, and how these connections alter in response to experimental interventions or illness (Friston, 2009).

Functional connectivity is defined as the temporal correlations among spatially distinct neurophysiological events (Büchel and Friston, 1997), which is traditionally calculated using correlation or partial correlation. Functional Connectivity (FC) in the brain is defined as a statistical link between the activation signals of two brain regions, after removing the influence of all other areas (Tognoli, 2014). Undirected functional connectivity (FC) measurements and directed effective connectivity (EC) metrics are the two types of connectivity measures, and they could be well tested on the fMRI data. Static FC is a common name for the correlation between brain voxels or regions during the whole duration of the scanning session. On the other hand, dynamic functional connectivity (FC) refers to non-instantaneous connections throughout time-series from a set of brain areas (Park et al., 2021).

The activations of brain areas may be correlated; however, these correlations are sometimes only a statistical outcome, whereas in certain circumstances this is due to the causal influence of one brain region over another. In other words, the brain areas may show excitatory or inhibitory effects on each other (Danks and Davis, 2023). Despite the properties of the fMRI, including low temporal resolution, and low signal to noise ratio, estimation of causality between the brain areas and during fMRI is an established and active field of research, and several models have been developed to address this challenge (Bielczyk et al., 2019).

Effective connectivity is defined as one neural system’s impact over another (Büchel and Friston, 1997). Because it tracks the direction of information flow throughout time, EC is inherently directional (Bielczyk et al., 2019). To evaluate effective connection, a model of how one region influences on another is required. Analyses of effective connectivity then attempt to quantify coupling in terms of the connectivity model’s characteristics. The two most utilized methods for estimating the EC between brain areas are the DCM (Dynamic Causal Modeling) (Friston et al., 2003) and GCM (Granger Causality modeling) (Goebel et al., 2003; Valdes-Sosa, 2004), which both appeal to causation and rely on time-series models of fMRI data. DCM attempts to model how activity in one brain area is affected by activity in another, whereas GCM seeks the signature of these influences by looking for correlations in the activity of two or more regions over time. The models used by DCM are more complex and domain-specific, but the GCM is more straightforward and generic, and is built under the assumption that any statistical dependencies across brain areas may be approximated by a (usually linear) mapping over time lags.

The brain uses a surprisingly high amount of energy, even at rest; it is reported that the brain uses 60–80% of its total energy for intrinsic activity, or communication between neurons and the cells that support them, and just 0.5–1% for evoked activity (Raichle and Mintun, 2006). For almost 50 years, philosophers have debated brain states, but no one has provided a clear description of what they are (Brown, 2006). Brain states are synchronized neuronal firing patterns that reflect the electrical face of the brain, and there is evidence on a wide range of brain states through distinct patterns of activity. The state of brain activity correlates to various degrees of consciousness, and therefore customized variants of the networks produce wakefulness, REM sleep, slow-wave sleep, various types of anesthesia, and other unresponsive states.

The intrinsic dynamics of the brain in the absence of any sensory or cognitive stimulus, which may be quantified as spatially and temporally segregated networks, are created by spontaneous brain activity (Deco et al., 2011). Several different networks usually manifest in the human brain during resting-state, and some of them are well known; examples include the salience network, auditory network, basal ganglia network, higher visual network, visuospatial network, default mode network, language network, executive network, attentional network, precuneus network, primary, ventral, and dorsal visual networks, and sensory motor network (Nishida et al., 2015; Smitha et al., 2017). The seed-based approach and independent component analysis (ICA) are the two main computational methods used to find such RSNs.

It is important to note that the brain’s networks during resting state are constantly changing. In other words, not all RSNs remain coherent for several minutes (Damoiseaux et al., 2006), and there are reports that they can be activated simultaneously or one at a time, and then they can also become deactivated and make way for other networks. These network shifts between brain states affect activities of the brain, and they are associated with the appearance of different cognitive functions. The dynamic switches between brain states have been shown in resting state acquisitions (Meer et al., 2020), and even some studies have illustrated that the temporal dynamics of brain states are reshaped during some active tasks, such as movie viewing (Meer et al., 2020). The spontaneous dynamics of the brain modulate its function from moment to moment, shaping neural computation and cognition; functional MRI, while classically used as a tool for spatial localization, is increasingly being used to identify the temporal dynamics of brain activity. The alteration of brain states during conditions is well illustrated; however, much less is known on the causal influences on this pattern.

The human brain functions using both bottom-up and top-down mechanisms. Bottom-up processing involves stimulus shaping perception, while top-down processing uses background knowledge and expectations. Soma major functions such as visual selection (Theeuwes, 2005), attention allocation (Folk et al., 1992), and working memory content (Olivers et al., 2006) follow either mechanism. Some studies suggest an integration of both mechanisms, with early bottom-up processing followed by later top-down processing (Hochstein and Ahissar, 2002). Similarly, the intrinsic activities of the brain during resting state which results in the alteration of the networks could follow one of those two mechanisms; it might be the subcortical brain regions which are more influential over the cortical regions (being interpreted as a bottom-up approach), or the cortical areas being more causal over the subcortical brain regions (a top-down mechanism).

The predictive-coding model highlights the interdependence of bottom-up and top-down processes, while both theoretical reasoning and empirical data have presented challenges to the bottom-up and top-down paradigms. For example, in Tscshantz et al. (2023) it is mentioned that the debate on top-down and bottom-up signals in visual perception persists, due to conflicting findings. Also, the brain’s processing strategies shift adaptively between bottom-up and top-down dominance based on task demands and environmental context (Engel and Fries, 2010). It is also necessary to mention that bottom-up and top-down processes dynamically interact, creating feedback loops that are challenging to model theoretically (Friston, 2005). According to a study (Rauss and Pourtois, 2013), predictive coding (and active inference) frameworks do not assume that neural pathway directionality must match an association with bottom-up or top-down processes. Predictive coding in neocortex areas uses a hierarchical model learned from sensory inputs to understand perception, action, and neocortical architecture. The Rao-Ballard model suggests cortical circuits use Bayesian inference, with predictions of lower-level activities transmitted via top-down feedback loops. In turn, the feedforward, bottom-up connections communicate the discrepancies between the actual activities and the top-down predictions (Jiang and Rao, 2022). Consequently, in this context, it could be said that our hypothesis can be included in the bottom-up connections of the brain, but more studies and researches are definitely needed.

The dynamic nature of brain networks is crucial for various cognitive functions, including learning, memory, attention, and adaptation to new environments (Bassett and Sporns, 2017). As a result, this is important to fully understand the brain’s neuroplasticity, which is its ability to reorganize itself in response to various factors (Mateos-Aparicio and Rodríguez-Moreno, 2019). This is especially important to study when the brain is at rest and not involved in a particular task. As a result, our aim in this study was to identify the causal drivers of distributed activity during the resting state. In other words, there should be a cause for this pattern of alteration. We hypothesized that some of the brain activation networks of the resting state should themselves be influential in the switching of the brain states during rest. To address this aspect of functional integration, we collected resting state fMRI data using a 3 T MRI scanner and a 64-channel head coil from 64 young individuals who were meticulously checked for their mental and physical health. Using robust data analysis methods and utilizing the Granger Causality approach, we estimated the causality of each brain state on all other resting state networks, in search of the higher causal networks. The switching of the brain states takes place while the brain is at rest and partially subconscious, and this is related to a phenomenon known as unconscious free will. This area has a complex basis of philosophy, and we hope our endeavor here could partly reveal the mysteries of brain states dynamics during resting state.

## 2 Methods

### 2.1 Participants

The Iranian Brain Imaging Database (*IBID*) (Batouli et al., 2021) was established to enable the study of human brain function, assist clinicians in researching disease diagnosis, and connect Iranian researchers with an interest in the brain. Its goal was to provide a standard MRI data set of physically and mentally healthy participants across different age groups, and to develop a database of brain MRI along with cognitive tests. Multiple MRI protocols and numerous cognitive tests, mental health, lifestyle, and clinical assessments were performed on over 300 individuals from age 20 to 70 years old, with an equal number of participants (#60) for each decade of age. Each participant’s physical health status was clinically assessed by three different general practitioners, based on published criteria (Sah and Sisakhti, 2020), and each participant completed the assessments on two consecutive days. The ethical approval code for this study was IR.NIMAD.REC.1396.319, issued by the National Institute for Medical Research Development, in agreement with the Declaration of Helsinki, and informed consent was obtained from all participants.

In our study here, we used the data of group 1 of *IBID* dataset that included 64 subjects (33 male and 31 female) between 20 and 30 years old. For each subject, one T1 weighted image and one fMRI timeseries in the resting state was used.

### 2.2 Imaging

The MRI machine used in this study was a Siemens 3.0 Tesla scanner (Prisma, 2016), devoted to research, at the Iranian National Brain Mapping Lab.^1^ A few characteristics of this machine included 50-cm FOV with the industry best homogeneity; whole-body; superconductive zero helium oil-off 3 T magnet; and head/neck 20 direct connect. We used a 64-channel head coil in our study. The MRI protocols were selected to match the international projects, such as the UK Biobank or the ENIGMA consortium. The MRI protocols were as follows:

#### 2.2.1 Resting-state fMRI

Total time = 6 min; TR = 2,500 ms; Time-points = 144; TE = 30 ms; flip angle = 90 degrees; voxel size = 3.0 × 3.0 × 3.0 mm; #slices = 40; matrix size = 64 × 64 × 40; distance factor = 0%; phase encoding direction = anterior > > posterior; averages = 1; delay in TR = 0 s; multi-slice mode = Interleaved.

#### 2.2.2 T1-weighted MP-RAGE

TA = 4:12 min; TR = 1800 ms; TE = 3.53 ms; TI = 1,100 ms; flip angle = 7 degrees; voxel size = 1.0 × 1.0 × 1.0 mm; multi-slice mode = sequential; FOV read = 256 mm; #slices = 160; phase encoding direction = anterior > > posterior; matrix size = 256 × 256 × 160; averages = 1.

### 2.3 Quality check and preprocessing

All MRI data were visually checked for good quality, based on previous methods (Sisakhti et al., 2021, 2022). This step included image information such as matrix and voxel sizes, the number of time-points (for resting-state fMRI), and checking the images to be right-to-left oriented. Besides, the visual check was performed to spot possible macroscopic artifacts and vibration/motion evidence in images and to check head tilt and head positioning, signal loss, ghosting, or other possible artifacts in the data. During the visual check, one male participant was excluded from the dataset, as his fMRI data was inaccurately collected. This resulted to including 63 participants in total (32M and 31F).

### 2.4 fMRI data analysis

**Figure 1:**
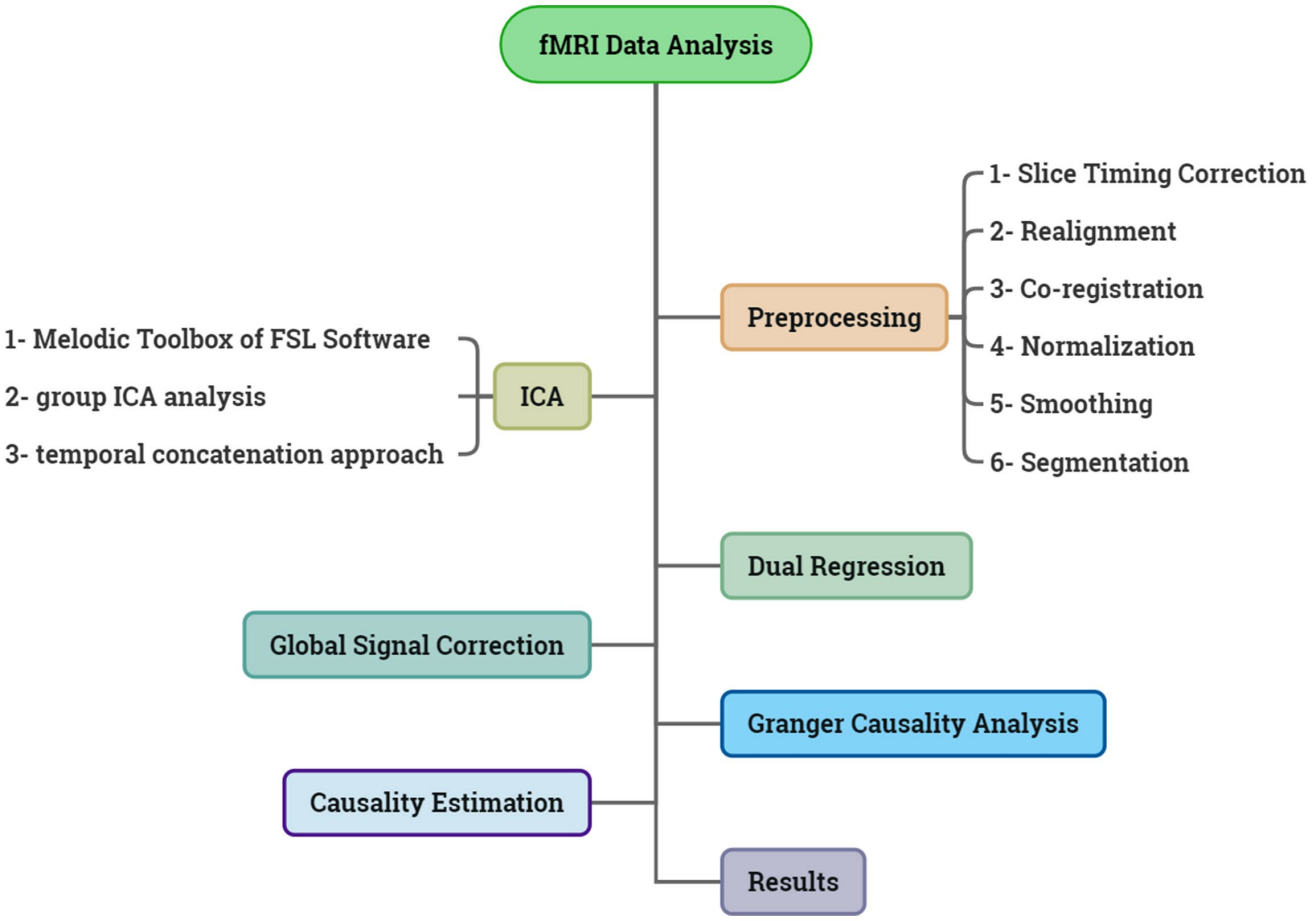
Summary of the steps taken in this article.

In summary, the stages employed in this article are depicted in the Figure 1.

#### 2.4.1 Preprocessing

We performed six steps of preprocessing in SPM 12 (statistical parametric mapping, last updated 13th January 2020) on the fMRI data, including slice timing correction, realignment, co-registration, normalization, smoothing, and segmentation. These steps were selected based on the pipeline used in the UK Biobank (Alfaro-Almagro et al., 2018). In slice timing section, the following settings were applied: number of slice = 43; TR = 2,500 ms; TA = 0.9768 (1–1/43). For realignment, the settings were: quality = 0.9; separation = 4; smoothing =5; and interpolation =5. The settings of co-registration included: for reference image, we chose T1 image and for source image, and all volumes of the resting state images were chosen. In normalization step, for the image to align we selected the T1 image, and for image to write, we selected all volumes of the resting state images that were extracted from the last preprocessing step (co-registration). The setting of smoothing was: FWHM = 6; data type = same; implicit masking = none.

The final preprocessing step was performed on the T1-weighted image. Since fMRI studies focus on brain tissue, in this step, we removed the skull and non-brain tissues from the T1-weightedbrain image. FSL (FMRIB Software Library v6.0 Created by the Analysis Group, FMRIB, Oxford, UK.) has a tool for this called BET (Brain Extraction Tool), and we used BET GUI in FSL with these settings: fractional intensity threshold = 0.35; bias field and neck cleanup.

#### 2.4.2 Melodic ICA

We used the MELODIC toolbox (Multivariate Exploratory Linear Optimized Decomposition into Independent Components), FSL software package (FMRIB Software Library v6.0 Created by the Analysis Group, FMRIB, Oxford, UK.), in order to identify the brain activation maps during the resting state; these brain activations are referred to as independent components in the spatial ICA algorithm performed in Melodic, FSL. Independent Component Analysis is used to decompose a single or multiple 4D data sets into different spatial and temporal components.

The preprocessed data were imported into MELODIC (group ICA analysis, temporal concatenation approach), in order to pick out different activation and artifactual components without any explicit time series model being specified. The settings of the MELODIC analysis included: number of inputs = 63; slice timing correction = interleaved; motion correction = MCFLIRT; spatial smoothing FWHM = 5 mm; activate intensity normalization; multi session temporal concatenation mode of analysis; and Threshold IC maps = 0.9. The temporal concatenation approach resulted in 114 independent components for all the 63 fMRI datasets.

#### 2.4.3 Dual regression

Dual regression is a tool that we can use as part of a group-level resting state analysis to identify the subject-specific contributions to the group level ICA. The output of dual regression is a set of subject-specific spatial maps and time courses for each group level component (spatial map) that can then be compared across subjects/groups. As a result, in this way, we can extract the signal of each component for each subject.

All steps of dual regression were applied in FSL software. We applied dual regression on the outputs of the MELODIC ICA by a very simple code in the virtual machine of Linux in the WINDOWS environment. The Dual Regression coding was applied on the outputs of the MELODIC ICA step, where there were 114 components estimated for all the 63 participants together; however, by the end of this stage, a matrix with a size of 144×114 was created for each subject (144 rows correspond to the number of fMRI data volumes and 114 columns correspond to the number of components extracted from the MELODIC ICA).

#### 2.4.4 Effective connectivity

The matrix of Granger causal connectivity was estimated separately for each of the 63 participants. For each individual, we had a matrix with a size of 144×114, and in this matrix, obtained from dual regression, we separated the columns. Thus, for each subject, we had 114 column-wise matrices with dimensions of 144*1. These 114 matrices for each subject, resulting in a total of 63×114 fMRI signals, served as input for effective connectivity estimation.

Our hypothesis in this study was that the brain networks active during the resting state have causal influences on each other, and in this study we are trying to find the networks with the highest causality effects. As a result, for each individual, the output would be a matrix with the size of 114 × 114, and each row of the matrix, for example row 5, is showing the causal influences of component 5 on all other components. A graphic illustration of our proposed model is provided below:

**Table:** 

$\begin{array}{cccccccccccc} {\mathrm{component}}_{1} & \underset{\to }{\mathrm{Granger Causality Analysis}} & {\mathrm{cause}}_{1} & = & \overset{\mathrm{number of components}=N}{\underset{\alpha =1}{\sum }} & \alpha_{1}.\left({\mathrm{component}}_{1}\right) & + & \alpha_{2}.\left({\mathrm{component}}_{2}\right) & + & \ldots & + & \alpha_{N}.\left({\mathrm{component}}_{N}\right)\\ {\mathrm{component}}_{2} & \underset{\to }{\mathrm{Granger Causality Analysis}} & \;{\mathrm{cause}}_{2} & = & \overset{\mathrm{number of components}=N}{\underset{\alpha =1}{\sum }} & \alpha_{1}.\left({\mathrm{component}}_{1}\right) & + & \alpha_{2}.\left({\mathrm{component}}_{2}\right) & + & \ldots & + & \alpha_{N}.\left({\mathrm{component}}_{N}\right)\\ . & . & . & & . & . & & . & & \ldots & & .\\ . & . & . & & . & . & & . & & \ldots & & .\\ . & . & . & & . & . & & . & & \ldots & & .\\ {\mathrm{component}}_{N} & \underset{\to }{\mathrm{Granger Causality Analysis}} & \;{\mathrm{cause}}_{2} & = & \overset{\mathrm{number of components}=N}{\underset{\alpha =1}{\sum }} & \alpha_{1}.\left({\mathrm{component}}_{1}\right) & + & \alpha_{2}.\left({\mathrm{component}}_{2}\right) & + & \ldots & + & \alpha_{N}.\left({\mathrm{component}}_{N}\right) \end{array}$

Estimation of causality was based on the Granger Causality algorithm, performed in the REST toolbox (The latest release is REST_V1.8) (Song et al., 2011) and GCA (Granger Causality Analysis) part of that in MATLAB. For each individual participant, 114 signals were imported into the software one by one in the REST GCA toolbox, and the settings of the software were as follows: ROI-wise mode = multivariate coefficient mode; masking = user defined mask; and order = 1. The outputs of this step for each individual were a 114 × 114 matrix that contained the positive and negative effective connectivity coefficients.

#### 2.4.5 Estimation of causality

In this step, the effective connectivity coefficients of the 63 individuals were combined, to find the components with the highest causality effects. Combining the results of individuals could be performed in three approaches: (I) to separate the positive and negative effectivity coefficients for each individual, and then sum them separately among all the individuals. In this approach the negative values represent an inhibitory effect and the positive values represent an excitatory effect; (II) to sum all the effectivity coefficients among the individuals, regardless of the sign; and (III) to take the absolute values of the elements of the matrixes, and then sum them together among the individuals. In this work we selected the third approach, as we speculated it could better show the causality effect of a component, and there were also some prior studies which used the absolute values when estimating the effective connectivity between the brain regions (Sanchez-romero et al., 2019; Zarghami and Friston, 2020; Shahabi et al., n.d.).

It is important to notice that the sum of the elements of each row is the sum of the effects of (for example) component #1 on the rest of the other components.

### 2.5 Global signal correction

Our estimations so far had not considered the effects of the fMRI Global Signal on the signals of each resting-state component. The Global Signal (GS) is the average of the time courses of all gray matter voxels (Li et al., 2019). We repeated our analysis by removing the effects of the GS on the components, and it is important as the GS effects may be mistakenly considered as a causal factor. This correction is called global signal regression (GSR) (Liu et al., 2017).

To explain how we performed the GSR, assume: Y = a.X + ε; “Y” is the signal of each of the 114 components, “a” is a constant, “X” is the global signal of each subject, and “ε” is the residuals and the desirable signal of each subject after clear out of global signal effect. But before inserting signals in the above equation, we normalized the Y and X signals (converted to z-value), and accordingly the “ε” signal will be obtained as a normalized signal. It is well-known that to normalize a parameter, we perform the following equation: $z-\mathrm{value}=\frac{x-\mu }{\sigma }$ ; “x” is the observed value (raw score), “μ” is the mean of the sample, and “σ” is the standard deviation of the sample.

After calculating the normalized ε for each of the 114 components of each of the 63 individuals, it was converted back to the normal values, using this equation: $\varepsilon_{\mathrm{NEW}}=\left(\varepsilon_{\mathrm{normalized}}\right)\times \left(\sigma_{Y}\right)+\mu_{Y}$ ; “ $\varepsilon_{\mathrm{NEW}}$ “is our corrected signal for each component of each subject, $"{\varepsilon_{\mathrm{normalized}}}^{"}$ is the ε signal before GSR (normalized ε), ${\;}^{"}{\sigma_{Y}}^{"}$ is the standard deviation of each component of each subject’s signal and ${\;}^{"}{\mu_{Y}}^{"}$ is the mean of each component of each subject’s signal.

Now, and after obtaining the signals of the components after GS correction, we have 114 corrected signals for the 63 subjects, and we repeated all the steps above to estimate the causality coefficients once again. We used the REST GCA toolbox again, with the following settings: ROI-wise mode = multivariate coefficient mode; masking = user defined mask; and order = 1. The outputs of this step for each individual were a 114 × 114 matrix that contained the positive and negative effective connectivity coefficients.

We estimated the total causality effect of each component across all data; sorted the components based on the amount of causality, and selected the components with the highest effects. It should be noted that this process, yielded 12 causal networks.

### 2.6 Causality direction

In the above, we estimated which components showed the highest causality effects. In this step we wanted to identify the components which were mostly under the influences of those causal networks. For each causal network (12 networks in total), we sorted its effects on the other components, and its top 20% causality coefficients were selected. The choice of top 20% for thresholding the causality coefficients is based on a previous study (Zixiang et al., 2024). All components which met this criteria were selected, and they were regarded as the networks which were mostly under the influence of the causal networks.

### 2.7 The causality values

We identified 114 regions from our ICA analysis, and therefore we should have 114 values (sum of the absolutes) for the causality effects of each component on all others.

**Figure 2:**
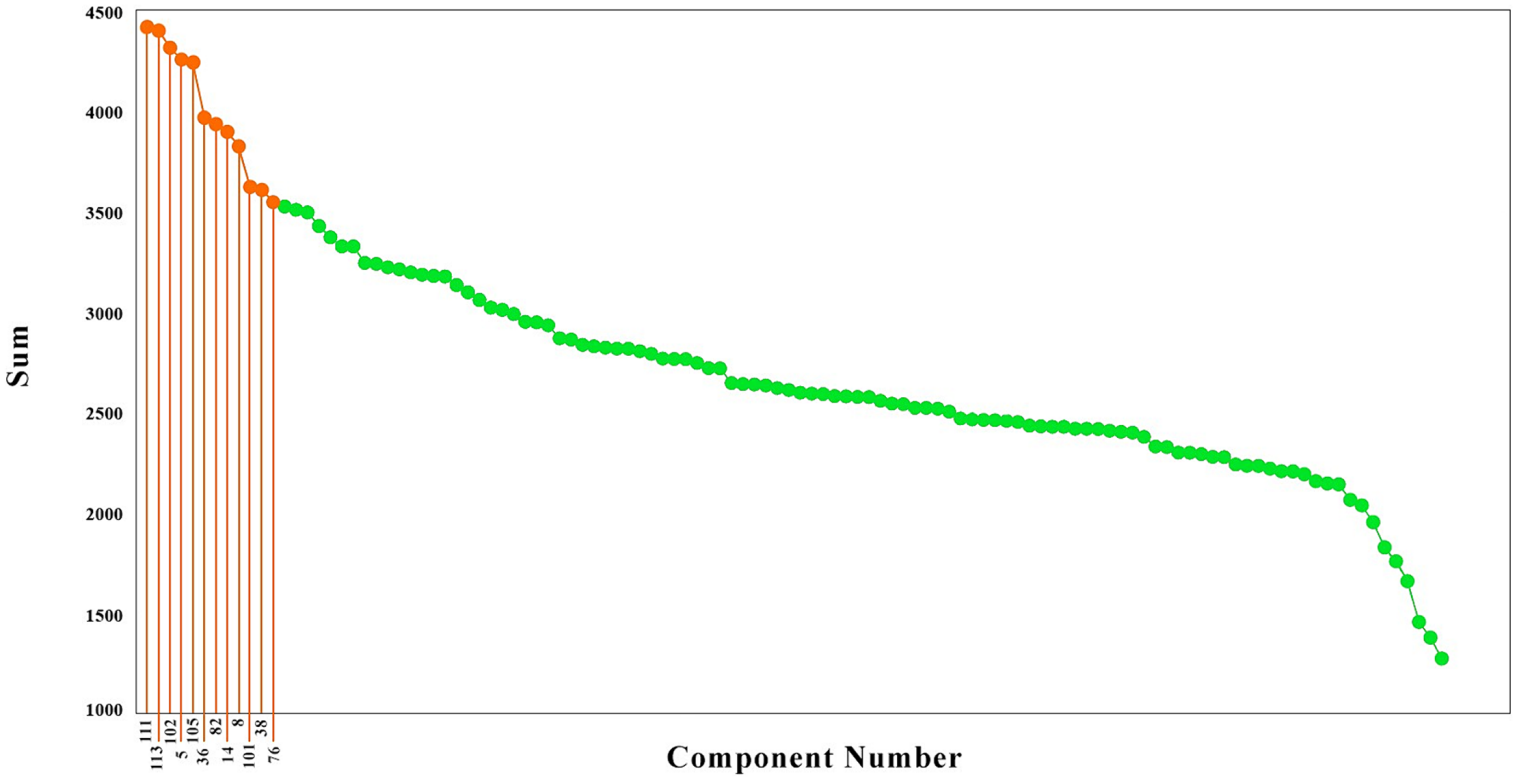
The brain components sorted based on their causality effects. The causality effects of the components are illustrated in green, and the components which had the top 20% causality effects are shown in orange. The numbers connected to the high causality components are the component numbers (That has been shown only for the top 20 percent).

To have a better idea of the components with the highest causality effect, we sorted the brain components from the higher causality effects to the lower, as illustrated in Figure 2. These components are illustrated in red color. As is observed, the causality effects vary among the brain components, with values ranging from around 1,300 (arbitrary values) to nearly 4,500. To choose the brain networks which are stronger in their causality, we selected the components with the top 20% causality effects. The highest causality coefficient was 4413.19, and therefore the components with a value above 3530.55 were selected among the top 20%. This resulted in 12 components, illustrated in blue in Figure 2, which included the components number 111, 113, 102, 5, 105, 36, 82, 14, 8, 101, 38, and 76.

### 2.8 The causal brain networks

**Figure 3:**
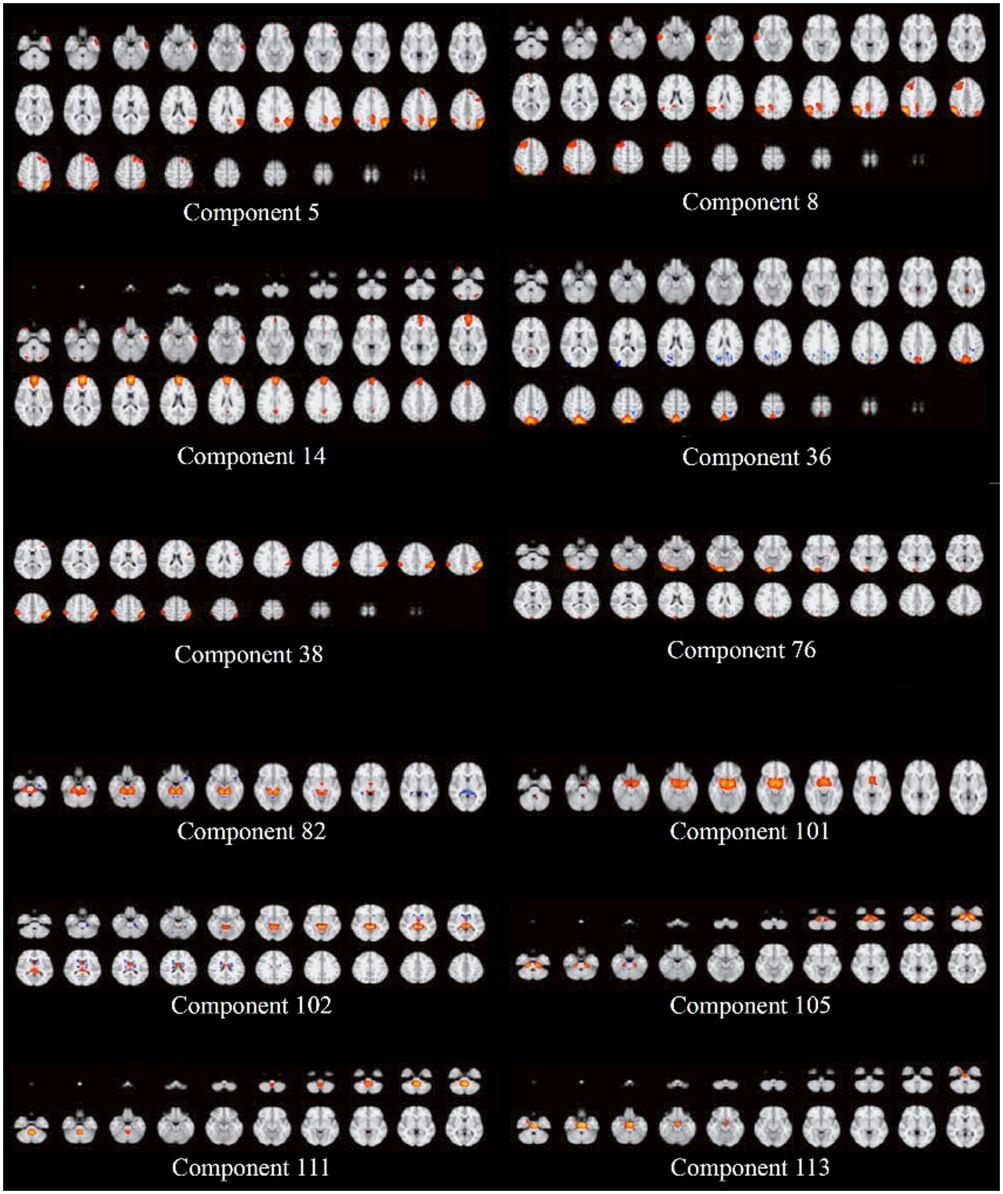
The activation maps of the components with the highest causality effects. There were 12 networks in the post-GS correction. Most networks included the subcortical brain areas, such as the brain stem, thalamus, amygdala, and posterior cingulate cortex.

The brain activation components with the highest causality effects, are illustrated in Figure 3.

However, to identify the main brain region being active in each component, we used the FSL eyes toolbox in the FSL software package, and tried to locate the core area of each component in the brain, using the standard atlases. These results are provided in Table 1.

**Table 1:** The components of the resting brain function with the top 20% causality values.

**Component number**	**Sum value**	**Main brain region**
111	4413.199227	Brain stem
113	4394.348625	Brain stem
102	4310.135885	Thalamus
5	4251.518294	Left lateral occipital
105	4236.337062	Brain stem
36	3961.601966	Precuneous
82	3930.660176	Brain stem
14	3891.952879	Frontal pole
8	3820.364736	Right lateral occipital
101	3618.445033	Amygdala
38	3603.503652	Supramarginal gyrus
76	3542.151380	Fusiform

After the GS-correction, it resulted in 12 networks. The sum values are the integrative causal values of each component on all other components. The main brain region relevant to each component is also provided.

As our GS corrected results are from a more robust approach, we mainly focused on those causal brain components, which included the brain stem, thalamus, lateral occipital, precuneus, frontal pole, amygdala, supramarginal gyrus, and fusiform. In the Melodic ICA analysis, some of the final components are relevant to the noises, subject motion, heart and respiratory rates, or other confounding factors; however, the main causal networks in our work were not from those undesirable components, which could serve as preliminary evidence supporting the reliability of our results.

## 3 Results

### 3.1 The networks under the causal influence

**Figure 4:**
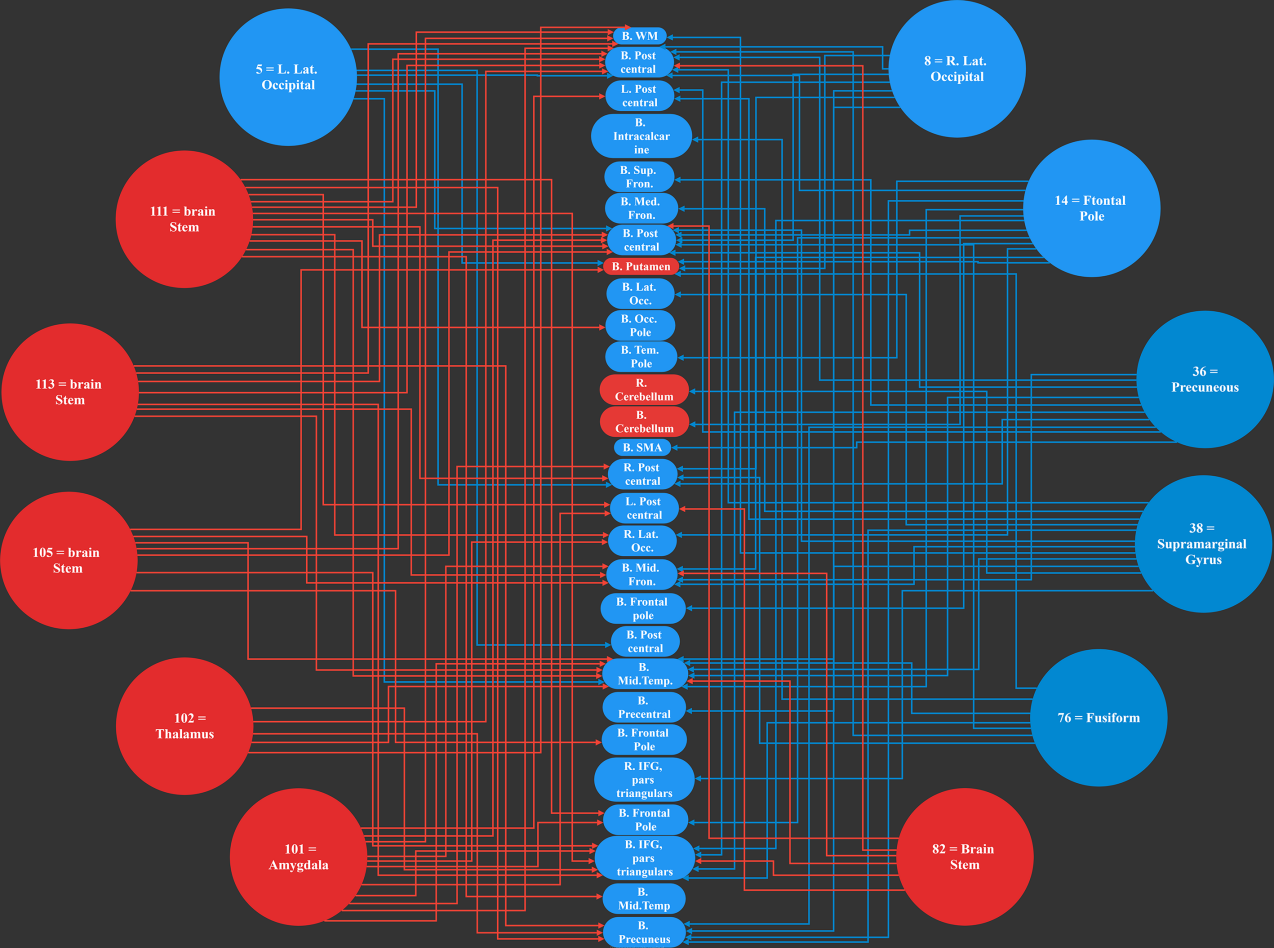
The 12 most causal brain networks are illustrated in circles, and the 28 mostly affected networks are illustrated in ellipses. All the blue circles and ellipses represent cortical regions, while all the red circles and ellipses represent sub-cortical areas; an arrow show a causal influence of the causal networks over the effect networks.

Our work resulted in identifying 12 main causal brain networks. However, a question arises that, on which brain networks do these causal networks are showing their influences? For this reason, we identified the networks being under those causal influences, and these results are illustrated in Figure 4. As is observed there, 28 networks were mostly under the influence of the causal networks. Next, we estimated the amount of influence on each of these “affected” networks. As provided in Table 2, the following brain regions were mostly under the causal influences: middle temporal, postcentral, inferior frontal, precuneus, and middle frontal gyri.

**Table 2:** The 12 higher causal networks are in the top row (C5–C113), and the amount of their causal influence on any of the 28 affected networks (provided in the first column) are provided in the table.

		**C5**	**C8**	**C14**	**C36**	**C38**	**C76**	**C82**	**C101**	**C102**	**C105**	**C111**	**C113**	
#C.	**Region**	Lat. Occ.	Lat. Occ.	Fron. Pole	Precuneus	Supra marginal Gyrus	Fusiform	Brain stem	Amygdala	Thalamus	Brain stem	Brain stem	Brain stem	**Sum**
c80	B. Mid. Temp.	58.57	50.32	51.55	54.78	43.05	55.04	55.69	43.02	60.87	53.94	56.50	51.90	635.28
c3	B. Post central	56.22	53.89	51.97	53.19	43.10	46.05	47.94	44.60	54.11	58.58	57.76	52.34	619.80
c16	B. Post central	46.90	52.43	49.59	45.51	46.13	44.18	46.14	45.29	--	59.39	60.36	58.59	554.56
c97	B. IFG, pars triangularis	--	47.72	46.87	45.10	--	47.70	45.29	42.58	48.98	57.8	52.39	55.77	490.23
c112	B. precuneus	--	45.78	44.01	53.09	37.56	--	--	--	51.79	--	53.91	48.70	334.88
c65	B. Mid. Fron.	--	--	46.89	45.93	42.80	--	47.14	38.94	--	51.50	48.90	--	322.12
c57	R. Post central	47.02	43.58	--	45.03	--	46.66	--	48.46	--	--	--	47.75	278.53
c1	B. WM	--	--	--	--	38.22	--	--	44.14	54.97	--	57.01	47.42	241.78
c27	B. Putamen	54.02	46.21	43.51	--	--	47.37	--	--	--	48.08	--	--	239.22
c60	L. Post central	--	--	--	--	--		51.35	43.55	--	--	--	52.21	147.11
c94	B. Fron. pole	--	--	44.60	--	--	--	--	39.02	--	--	--	55.97	139.60
c64	R. Lat. Occ.	--	--	42.44	--	--	--	--	41.46	--	--	--	49.36	133.27
c4	L. Post central	--	--	--	44.07	38.76	--	--	40.32	--	--	--	--	123.17
c106	B. Mid. Temp.	--	--	--	--	--	--	--	--	--	--	--	50.64	50.64
c66	B. Fron. pole	--	--	50.08	--	--	--	--	--	--	--	--	--	50.08
c39	B. Occ. Pole	--	--	--	--	--	--	--	--	--	--	--	49.72	49.72
c85	B. Fron. pole	--	--	--	--	--	--	--	--	--	49.02	--	--	49.02
c69	B. Post central	47.12	--	--	--	--	--	--	--	--	--	--	--	47.12
c6	B. Intracalcarine	--	--	--	--	--	44.94	--	--	--	--	--	--	44.94
c81	B. Precentral	--	--	--	--	44.75	--	--	--	--	--	--	--	44.75
c40	B. Tem. Pole	--	--	44.57	--	--	--	--	--	--	--	--	--	44.57
c11	B. Sup. Fron.	--	--	--	44.32	--	--	--	--	--	--	--	--	44.32
c52	B. SMA	--	--	--	43.99	--	--	--	--	--	--	--	--	43.99
c50	B. Cerebellum	--	--	42.52	--	--	--	--	--	--	--	--	--	42.52
c49	R. Cerebellum	--	--	--	--	40.00	--	--	--	--	--	--	--	40.00
c29	B. Lat. Occ.	--	--	--	--	38.84	--	--	--	--	--	--	--	38.84
c12	B. Med. Fron.	--	--	--	--	38.05	--	--	--	--	--	--	--	38.05
c86	R. IFG, pars triangularis	--	--	--	--	37.92	--	--	--	--	--	--	--	37.92

The main brain region associated with any of the affected components is also provided in the second column. The final column shows the sum of all causal influences on each activation component. The values are arbitrary, and are the outputs of the software package. C, activation component number; B, bilateral; R, right; L, left; Mid, middle; Temp, temporal; IFG, inferior frontal gyrus; Fron, frontal; WM, white matter; Lat, lateral; Occ, occipital; Sup, superior; SMA, supplementary motor area; Med, medial.

This is interesting that, in our results, most of the causal networks were relevant to the deep structures of the brain, and on the other hand, most of the affected regions were in the cortical areas.

## 4 Discussion

### 4.1 Summary of the results

In this study we aimed to identify the brain areas which had the highest causality effects on the other brain regions during the resting-state. By integrating a number of previously established methods on estimating causality in fMRI data, we proposed a novel method for the analysis of causality in resting state fMRI, and observed that the highest causal brain regions were brain stem, thalamus, lateral occipital, precuneus, frontal pole, amygdala, supramarginal gyrus, and fusiform, distributed over 12 resting-state networks (Table 1). The regions with the highest causality effects were observed to be the subcortical regions, as the sum of their causality coefficients were about 24903 (arbitrary values), while the cortical regions had a lower sum of 23071. On the other hand, when considering the areas mostly being under the effect (Table 2), among the 28 functional networks diagnosed as being the highest impressionable, 24 areas were cortical regions, and only 4 networks were from the subcortical areas. As a result, the dominant finding of our work is a causal influence of the deep brain areas over the cortical regions of the brain during the resting-state.

### 4.2 The causal brain networks

We identified that some subcortical brain areas showed a causal influence over the cortical regions. One of the major influences here was from the brain stem. Similar findings are observed in previous works. One study discovered widespread negative connections between the cortex and all three brainstem nuclei including locus coeruleus, vental tegmental area, and substantia nigra, as well as positive correlations between activity in these nuclei and the activity in other subcortical locations (Büchel and Friston, 1997). In another study, the peaks in the global signal coincided with the brain stem function (Tognoli, 2014), which is consistent with some other reports which discovered a negative connection between the brain stem and cortical regions (Park et al., 2021), or discovered positive associations between physically defined brain stem subdivisions and some cortical targets (Danks and Davis, 2023). It is also reported that the brainstem has an effective connectivity on cortical regions (Valdes-Sosa, 2004), as well as reports on the brain stem influencing the motor learning (Raichle and Mintun, 2006).

One study suggested that the correlation between the intrinsic activity of the brain stem and the cortical areas is probably due to shared fluctuations across neuromodulatory nuclei; in other words, the neuromodulatory nuclei’s fluctuating activity drives several cortical regions, potentially influencing the intrinsic activity correlations within the cortex (Friston, 2009). Five significant neuromodulatory systems have been found in the brain: The locus coeruleus and the A1/A2 brainstem regions release norepinephrine; the substantia nigra and the ventral tegmental area are the sites of dopamine release; neurons in the basal forebrain emit acetylcholine; serotonin is produced by the raphe nuclei; and histamine is secreted by the hypothalamic tuberomammilary nucleus, which projects to nearly the whole forebrain (Friston, 2009). It is proposed that the changes in the activity of neuromodulatory nuclei correspond with the fast fluctuations in activity state. The activation of ionotropic receptors by serotonin or choline is thus a way by which the neuromodulatory brainstem system can rapidly shift the cortical activity state. As a result, the neuromodulatory brainstem systems can influence cortical population activity via a variety of mechanisms, including fluctuating brainstem nuclei driving large regions or subcortical regions and indirectly modifying cortical dynamic state (Friston, 2009).

In addition to brain stem, thalamus also showed significant causal influences over cortical regions. There are reports on the association of the thalamus with the other brain structures, such as between the thalamus and the basal ganglia, dorsal prefrontal cortex, and the anterior cingulate cortex (Jagtap and Diwadkar, 2016). In a study (Wolff and Vann, 2019), it was proposed that rather than simply operating as relays, thalamic nuclei contribute to cortical functioning and higher-order cognition as well, including learning and memory as well as flexible adaptation. Other data that supports the idea of the non-relay role of the thalamus has recently emerged. For example (Halassa and Kastner, 2017; Schmitt et al., 2017) and (Nakajima and Halassa, 2017) emphasize the thalamus’s role in managing cortical connectivity in order to sustain rule representation. A causal link between thalamus and prefrontal activity and social dominance behavior was recently found, further emphasizing the importance of thalamic inputs for cortical processes (Zhou et al., 2017). Numerous investigations have been conducted to demonstrate the relevance of thalamic inputs in driving activity in their cortical destination (Mathiasen et al., 2017). Indeed, the tight functional correlation between the thalamus, hippocampus, and retrosplenial cortex suggests that the thalamus may be important in coordinating activity across various regions (Corcoran et al., 2016; Halassa and Kastner, 2017).

Another thalamic nucleus that has drawn a lot of attention is the reuniens nucleus, which connects the prefrontal and temporal lobe regions by way of various frontal areas, and serves as the primary thalamic afferent to the hippocampus. This area is believed to be a significant hub, organizing functional exchanges between frontal areas and the hippocampus, especially in the absence of direct inputs from the prefrontal area to the hippocampus, as these projections are reciprocal (Wolff and Vann, 2019).

Precuneus also showed causal influences on other brain structures in our results. A study (Hillebrand et al., 2016) showed that in alpha-2 frequency band, precuneus had one of the strongest outgoing connections with the areas receiving these connections which were mostly cortical areas, such as the superior frontal gyrus, inferior frontal gyrus, supramarginal gyrus, anterior cingulate, and temporal pole.

There are other findings on the causality of the subcortical areas over the cortical regions; examples include amygdala and hippocampus on the ventrolateral prefrontal cortexes (Velichkovsky et al., 2019), amygdala and dorsolateral prefrontal cortex having a significant negative interaction (Klein-Flügge et al., 2020), amygdala having a causal effect on default mode network because of its significant connections with ventromedial PFC (Kerestes et al., 2017), right and left thalamus having a causal effect on the right and left cortices, respectively (Fasoula et al., 2013), and hippocampus and parahippocampus on the inferior parietal, superior frontal, middle temporal, and inferior frontal gyri (Park et al., 2018).

Although the major finding in our work was a causal effect from the deep brain regions to the cortical areas, we also observed causalities between the cortical areas. There are similar findings in previous works, such as the medial prefrontal region showing causality on the inferior parietal and inferior temporal areas (Friston, 2009), supplementary motor area having considerable connections to both left and right primary motor cortex (Jiao et al., 2014), and medial prefrontal cortex having a significant connection to the posterior cingulate cortex and inferior parietal lobules in the default-mode network.

Despite the reports on the one-way causal influences of some brain regions over others, there are reports that these connections could be reciprocal; in other words, the two brain regions mutually showing influences on each other. For example, in a resting state study, bidirectional connections between the posterior cingulate and parietal areas, and between the medial prefrontal and parietal areas were seen (Wu et al., 2011; Zhou et al., 2011). In another work (Yusoff et al., 2018), estimating the effective connectivity between the inferior parietal and inferior temporal gyri showed that they had a dynamic cooperation, the intrinsic connections between them were negative in both directions, and in other words, they were mutually inhibiting each other. Also, in Deshpande and Hu (2012) it was discovered that two areas, the posterior cingulate and inferior parietal regions, had mainly bidirectional connections with all other ROIs in the four networks of default-mode, dorsal attention, hippocampal cortical memory, and fronto-parietal control networks.

Our results attribute a low causality of the cerebellum in the modulation of cortical activity. To address this issue, we should mention that while the cerebellum is acknowledged for its role in enhancing the coherence of neuronal oscillations, its function is often described as coordinating communication between cortical areas rather than directly influencing cortical activity. This suggests a more indirect modulatory role, emphasizing coordination over direct causality in cortical modulations (McAfee et al., 2022). In a study (Streng et al., 2018) it is addressed that causality of cerebellum in cortical activity modulation is indirect, or it is also (Popa and Ebner, 2019) mentioned that indirect causality of cerebellum influences cortical activity in prediction-error processing. Similarly, another study depicted that cerebellum modulates cortical activity through feedback/feed-forward prediction-error processing (Peng et al., 2021).

### 4.3 Effective connectivity estimation

#### 4.3.1 Mathematical models

To estimate the effective connectivity, a model of how one brain region influences another region is required. Both the two mostly used models, the DCM (Friston et al., 2003) and GCM (Goebel et al., 2003; Valdes-Sosa, 2004), appeal to causation and rely on time-series models of fMRI data. There are also a number of other methods for the estimation of the effective connectivity. The Tigramite (time-series graph-based measures of information transfer) is one example, and it is based on conditional independence testing under some assumptions, such as time-order, causal sufficiency, the causal Markov condition, and faithfulness (Runge, 2018; Saetia et al., 2021). Because time-lag is included, this framework may demonstrate changes in the causal model over time, which is important for pathway inference (Mannino and Bressler, 2015). Another model, the Transfer Entropy (TE), is a model-free method for detecting the directed information transfer (causality) between stochastic processes (Saetia et al., 2021), and conditional mutual information (CMI) (Hlaváčková-Schindler et al., 2007) in the form of TE (Schreiber, 2000) is the information-theoretic function used in this approach.

Dynamic causal model measures effective connectivity because it considers how underlying neuronal processes affect each other. The related procedures in some applications of GCM rest upon blind deconvolution to deconvolve the observed BOLD signal into an underlying neural time series (David et al., 2008; Ryali et al., 2011; Sathian et al., 2013; Wheelock et al., 2014; Hutcheson et al., 2015; Goodyear et al., 2016; Ryali et al., 2016) that enables the estimation of effective connectivity. Based on a proposed distinction (Pedro et al., 2011), methods can be classified based on their approach to the temporal sequence of the samples, and one category is based on the temporal sequence of the signals [e.g., Transfer Entropy (Schreiber, 2000), or Granger Causality (Granger, 1969)], whereas others, such as Bayesian Nets (Frey and Jojic, 2005), do not pull information from the time sequence and instead rely solely on the statistical features of the time series. The GCM contains a multivariate approach that searches for directed graphs without imposing any particular structure onto the graph, and therefore these methods will be referred to as network-wise models (Seth et al., 2015).

We used the GCM model in our study, and there are evidences on the robustness of this approach. Some of the advantages of the GCM include: Granger causal models consider lagged links (Waldorp et al., 2011); GCM entails autoregressing a group of time series variables to determine which variables predict the values of which other variables most directly (Granger, 2008; Friston et al., 2014); GCM is employed as a model-free method, requiring no strong assumptions about the structural connection underlying the specified ROIs; GCM begins with a complete graph spanning a large set of ROIs and gradually eliminates links between variables that do not reliably predict each other (Danks and Davis, 2023); also, if we want to anticipate future brain states based on the current condition, GCM is a powerful time series analytic tool (Hyung, 2001); GCM can be applied to either observed BOLD responses (Regner et al., 2016; Zhao et al., 2016; Chen et al., 2017) or deconvolved BOLD responses (David et al., 2008; Ryali et al., 2011; Wheelock et al., 2014; Goodyear et al., 2016; Ryali et al., 2016); and finally, the previous studies (Roebroeck et al., 2005; Seth et al., 2013) demonstrate that GCM is still instructive regarding the directionality of causal linkages in the brain (Seth et al., 2015). As a result, it seems that our findings which were based on the GCM could be reliable.

#### 4.3.2 Neural causality

In the brain and to perform a task, simultaneous events occur at the sub-neuronal, neural, and neuronal network levels. It is believed that because the processes at the various levels of explanation simultaneously occur, they are connected by a non-causal supervenient relationship whereas causality in brain best describes how it operates within levels but not between them. It is suggested that three requirements are needed for causality; first, interventionist causality conditions must be met; second, the occurrences that are causally connected should be explained at the same level; and third, a need for temporal order must be met, with an appropriate time scale on the order of 10 ms (Rolls, 2021).

(I) An interventionist account is widely accepted, suggesting that removing a potential cause and preventing the putative effect increases the likelihood of the potential cause causing the effect (Levels, 2020). (II) The argument suggests that causality operates within a level of explanation, not between levels. This means that cause and effect must be within the same level of explanation. This can refer to mental, computational, single neuron, or transmitter-influenced ion channels (Rolls, 2021). (III) Temporal order can be a useful criterion for identifying causality at the macro level of events in the mind, brain, and computers. In neuroscience, a time delay occurs when causes produce effects, allowing for Granger causality. In neuroscience, a time scale of 10 ms is sufficient for a causal event to be tested, as this time scale is similar to the time-scale of computation in the brain. This argument suggests temporal order is a useful criterion for causality in the brain (Rolls, 2021).

### 4.4 Neuroanatomical information and structural neuroimaging

Subcortical regions, such as the thalamus, basal ganglia, and brainstem nuclei, are integral to various neuromodulatory systems that influence cortical function. For instance, in (Sherman, 2016) it is mentioned the thalamus serves as a major relay station, transmitting sensory and motor signals to the cortex and playing a crucial role in consciousness, sleep, and alertness. Neuroanatomical studies have detailed extensive reciprocal connections between the thalamus and cortical regions, highlighting the thalamus’s role in integrating and modulating cortical activity. Another evidence is for basal ganglia that mentioned some structures such as the striatum and globus pallidus are involved in motor control, cognition, and emotion. These structures form loops with the cortex, facilitating the modulation of motor and cognitive functions (Haber, 2016). The next one is brainstem that brainstem nuclei, such as the locus coeruleus and raphe nuclei, which project widely to the cortex and influence cortical activity through the release of neuromodulators such as norepinephrine and serotonin. These projections play a critical role in arousal, attention, and mood regulation (Sara and Bouret, 2012).

Diffusion Tensor Imaging (DTI) studies also provide structural evidence supporting the connectivity between the subcortical and cortical regions. DTI studies have mapped the thalamocortical tracts, revealing the extensive white matter connections between the thalamus and various cortical regions, supporting its role in sensory and motor integration (Behrens et al., 2003). Also, DTI studies have identified the structural connectivity of corticostriatal tracts, providing evidence of the basal ganglia’s role in modulating cortical functions through these pathways (Lehéricy et al., 2013). DTI has also been used to map the brainstem’s projections to cortical regions, highlighting the structural underpinnings of the neuromodulatory influences from the brainstem to the cortex (Edlow et al., 2016).

### 4.5 Dynamic effective connectivity and clinical applications

It is crucial to note that a causal influence of the subcortical areas over the cortex is less studied in the literature. Most resting state effective connectivity studies focus on cortical regions, often neglecting the complex interactions between cortical and subcortical areas. As a result, performing studies focusing on the temporal dynamics of subcortical regions and their integration into cortical networks during different cognitive states are required (Hwang et al., 2017), which could have significant implications in the psychiatric and neurological disorders.

Human brain networks may be characterized by a system of interconnected brain regions that have been recognized by time-dependent observations via fMRI. To identify patterns, discover anomalies, and interpret temporal dynamics, it is critical to understand the changes of effective connectivity as a biomarker of neurodegenerative and psychiatric disorders, connections between different brain areas, and how these connections develop over time (Zhao et al., 2022). In DeMaster et al. (2022) it is demonstrated how alterations in resting state effective connectivity can serve as biomarkers for depression, or another work (Wang et al., 2022) highlights the changes in effective connectivity in schizophrenia, aiding in better diagnosis and understanding of the disorder. A study validates the use of dynamic causal modeling (DCM) for resting state effective connectivity, which can detect early connectivity changes in neurodegenerative diseases (Razi et al., 2015). In Bacon et al. (2023) it is investigated how resting state effective and functional connectivity can help to understand the network disruptions caused by interictal discharges. Another study discusses how connectivity can reflect network reorganization and recovery following brain injury (Nakamura et al., 2009). One research offers novel evidence about the pathophysiology of Autism in children by examining the effective connections within and between large-scale brain networks (Wei et al., 2022). As a result, studying the causality between the cortical and subcortical areas could also have clinical applications in the future.

### 4.6 Philosophical implications of the findings

One implication of our findings could be suggesting a hypothesis for the mind-brain interaction dilemma. A rough hypothesis is that, the brain areas which show the highest causality effects may be the place of interface between the mind and brain, albeit by having this assumption that the human mind is superior over the human brain. The issue of causality in the sciences of the mind and brain have always been under debate. The mind–body problem is primarily focused on how the mind and body can interact causally; specifically, how the mind can react to the body, and how the mind can control the actions of the body. The causal power of the mind is assumed as a thinking, rational entity, and all activities and productions, including mental and intellectual inventions, are attributed to it (Hookway, 1986; Batouli and Sisakhti, 2019).

Causality in the brain is the most important relationship that can help us to solve the mind-brain interaction. A mental event appears to cause a sequence of complicated and coordinated bodily motions, which have further downstream repercussions in the physical world (Harnad, 2000). It is the causal status of the mental component that lies at the core of the mind–body problem. The underlying neural mechanism causes both the brain’s and body’s functional neural/behavioral states and the fact that those states also happen to be mental states. This is known as third-party causation in the mind/body theory (Harnad, 2000).

Mental causation is at the heart of the mind–body problem. In particular, asking how mind and body interact is asking how they could impact one another. The sort of agency necessary for freewill and moral responsibility appears to necessitate mental causation (Harnad, 2000). There are researchers who have suggested the duality of the mind and brain. René Descartes introduced dualism into Western philosophy. Substance dualists, such as Descartes, believe that the mind is an independently existent substance (Descartes, 1998). However, if we remove the raw monistic approaches to explain the fundamental and conceptual differences between the attributes of mind and brain, the bilateral causal role of mind-brain in perception and volition needs to be explained. In the mathematical and accurate explanation of this interaction and the mechanism of this effect, quantum theories of consciousness are pioneering. For example, the Eccles-Beck theory delves into the influence of quantum probability on the exocytosis process of pyramidal neurons, and according to this theory, the mind, conceived as a distinct entity separated from the brain, exerts its influence on the brain by determining the result of this quantum process within the realm of intrinsic quantum probability, acting as a hidden variable (Eccles, 1994). The Penrose-Hameroff theory or orchestrated objective reduction theory (Orch-or theory) recognizes the mental states and consciousness to arise from quantum information processing at the level of neuron microtubules. They also propose a descriptive explanation of the causal role of the mind on the brain (Hameroff and Penrose, 2014). In addition, in an Avicenna-Bohm’s theory, which is a mathematical and physical explanation of the mind’s causal influences on the brain, it explains through the extended Bohmain quantum mechanics the role of the mind in determining the Bohmian force in guiding the nervous system toward the desire and imagination of the mind (Jamali et al., 2019).

If we do not deny the causal role of the mind in the brain, in addition to the mechanisms and physics of influence, the location of this interaction is of great importance. There are several theories about which part of the brain is related to the mind and mental states, and some of them propose that the whole brain is involving (Godwin et al., 2015; Jones, 2015; Cofré et al., 2020), and some of them have mentioned specific regions being responsible for it (Patrick, 2008; Zhao et al., 2019). Although today’s dominant cognitive science approach is that the cortex is responsible for the emergence of human cognition functions, this phenomenon may be under the control of deeper regions of the brain (Ward, 2013; Wolff and Vann, 2019). For example, special attention has been paid to the key role of thalamus in the emergence of cognitive issues and its role in the field of perceptual and dynamic control of cortex layer (Ward, 2013). The author in Ward (2011) suggested that primary conscious awareness is triggered by synchronized activity in dorsal thalamic nuclei’s dendrites, mediated by inhibitory interactions with thalamic reticular neurons, and suggests the thalamus’s anatomy and physiology play a central role in consciousness. Among the theories related to consciousness, Avicenna-Bohm’s theory emphasizes characteristically on the role of thalamus in the interaction between the mind and brain (Jamali et al., 2019), while Penrose-Hameroff’s and Eccles-Beck’s theories emphasize on the role of the sub-neuron levels and propose that this level plays a key role in the mind-brain interaction (Eccles, 1994; Hameroff and Penrose, 2014). However, due to the novelty of these theories and their mathematical and physical challenges, more studies are needed in this field in the future.

## 5 Conclusion

In this study we tried to identify the brain areas with the highest causality influences over other brain areas during the resting state. This was an endeavor toward numerous previous studies which tried to solve the interaction of the mind and body. During the resting state, numerous functional networks appear and alternate in the brain, and we hypothesized that, some brain areas which might be the places of interaction with the mind, should have causal influences in those alterations. We observed that the subcortical brain areas show a higher causality here, and the areas receiving those effects were mostly the cortical regions.

Despite our endeavors in selecting robust methodology for this study, there were some limitations with our work. First, we did not have the recordings for heart beat and respirations during the fMRI imaging. Although we used the ICA algorithm which is powerful in noise removal, it cannot be guaranteed that these physiological noises have not affected our results. Second, we used the Granger causality algorithm, which has advantages in some aspects, but utilizing other approaches could also provide confirmations for our findings. For example, accuracy of the Granger causality is known to be dependent on the TR, and the long TR in our study may cause errors. Third, our fMRI imaging had a time resolution of 2.5 s, and performing the imaging with a much better sampling rate is more robust in showing the dynamic aspect of functional integration in the brain at rest. Fourth is that 63 healthy adults in the age range of 20 to 30 years represented our study sample. Age has been found to both increase inter-network connectivity and decrease intra-network connectivity (Jones, 2015), and consequently, there is less potential of extrapolating our results to other populations. With the potential of rsfMRI to diagnose and track changes in brain function related to neurodegenerative disorders, this limitation is especially significant. Fifth, in this study, we used the absolute values of the effective connectivity matrix elements for each subject and summed them to achieve the highest degree of causal effect. However, as mentioned in the text, this can also be done by considering the signs of the elements, which might yield different results, and needs to be studied in the future. And finally, although we have suggested that our results could be a help to solve the mystery of the mind-brain interaction, as this is not clear today yet of what the human mind actually is and where this is located, the implication of our finding that the subcortical brain areas may be more in interaction with the mind is only a suggestion, and it needs further confirmation and studies in the future.

## Data availability statement

The original contributions presented in the study are included in the article/supplementary material, further inquiries can be directed to the corresponding author.

## Author contributions

OM: Methodology, Project administration, Software, Writing – original draft. GN: Conceptualization, Supervision, Validation, Writing – review & editing. SB: Conceptualization, Funding acquisition, Methodology, Supervision, Writing – review & editing.

## Ethics statement

The studies involving humans were approved by Iranian National Institute for Medical Research Development (Ethics Approval Statement: IR.NIMAD.REC.1396.319). The studies were conducted in accordance with the local legislation and institutional requirements. The participants provided their written informed consent to participate in this study.

## Footnotes

^1^ www.nbml.ir
